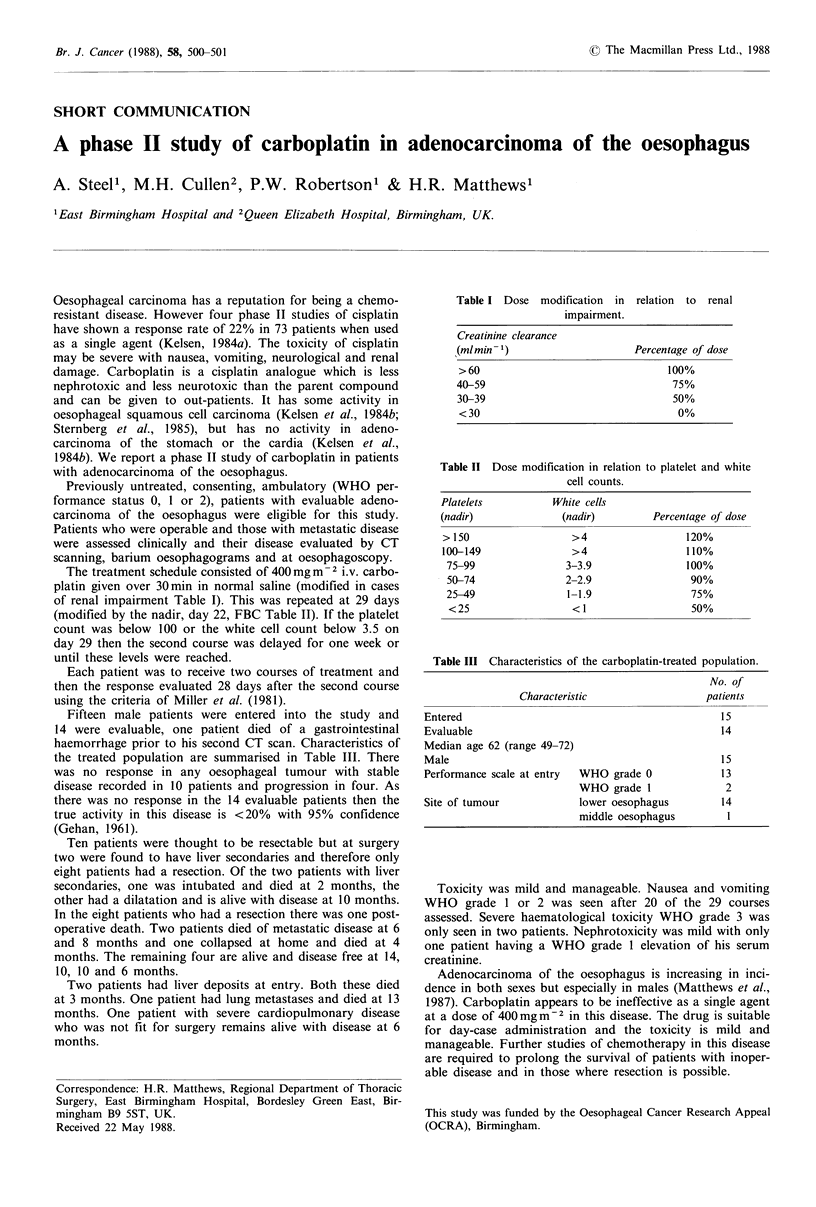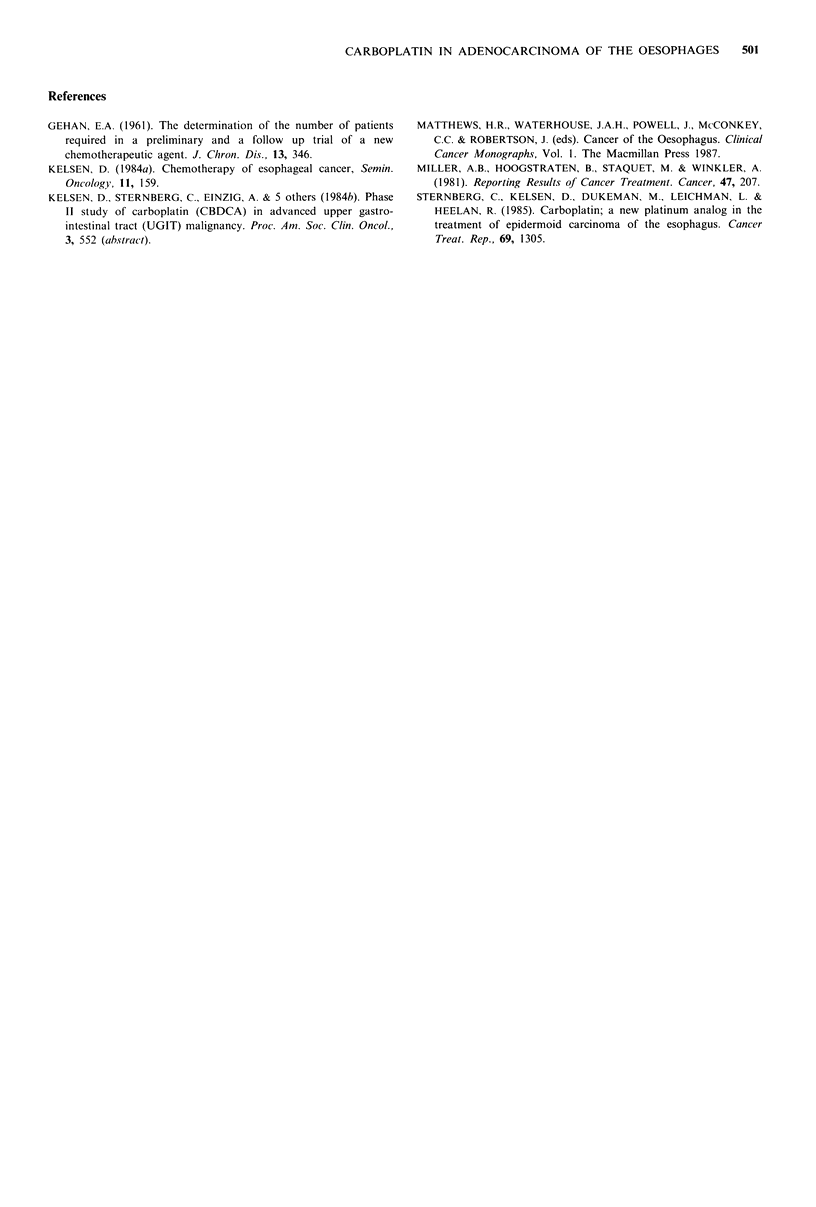# A phase II study of carboplatin in adenocarcinoma of the oesophagus.

**DOI:** 10.1038/bjc.1988.249

**Published:** 1988-10

**Authors:** A. Steel, M. H. Cullen, P. W. Robertson, H. R. Matthews

**Affiliations:** East Birmingham Hospital, UK.


Br. J. Cancer (1988), 58, 500–501

© The Macmillan Press Ltd., 1988

SHORT COMMUNICATION

### A phase II study of carboplatin in adenocarcinoma of the oesophagus

A. Steel[1], M.H. Cullen[2], P.W. Robertson[1] & H.R. Matthews[1]

[1]*East Birmingham Hospital and* [2]*Queen Elizabeth Hospital, Birmingham, UK.*

Oesophageal carcinoma has a reputation for being a chemoresistant disease. However four phase II studies of cisplatin have shown a response rate of 22% in 73 patients when used as a single agent (Kelsen, 1984*a*). The toxicity of cisplatin may be severe with nausea, vomiting, neurological and renal damage. Carboplatin is a cisplatin analogue which is less nephrotoxic and less neurotoxic than the parent compound and can be given to out-patients. It has some activity in oesophageal squamous cell carcinoma (Kelsen *et al.*, 1984*b*; Sternberg *et al.*, 1985), but has no activity in adenocarcinoma of the stomach or the cardia (Kelsen *et al.*, 1984*b*). We report a phase II study of carboplatin in patients with adenocarcinoma of the oesophagus.

Previously untreated, consenting, ambulatory (WHO performance status 0, 1 or 2), patients with evaluable adenocarcinoma of the oesophagus were eligible for this study. Patients who were operable and those with metastatic disease were assessed clinically and their disease evaluated by CT scanning, barium oesophagograms and at oesophagoscopy.

The treatment schedule consisted of $400\,mg\,m^{-2}$ i.v. carboplatin given over 30 min in normal saline (modified in cases of renal impairment Table I). This was repeated at 29 days (modified by the nadir, day 22, FBC Table II). If the platelet count was below 100 or the white cell count below 3.5 on day 29 then the second course was delayed for one week or until these levels were reached.

Each patient was to receive two courses of treatment and then the response evaluated 28 days after the second course using the criteria of Miller *et al.* (1981).

Fifteen male patients were entered into the study and 14 were evaluable, one patient died of a gastrointestinal haemorrhage prior to his second CT scan. Characteristics of the treated population are summarised in Table III. There was no response in any oesophageal tumour with stable disease recorded in 10 patients and progression in four. As there was no response in the 14 evaluable patients then the true activity in this disease is <20% with 95% confidence (Gehan, 1961).

Ten patients were thought to be resectable but at surgery two were found to have liver secondaries and therefore only eight patients had a resection. Of the two patients with liver secondaries, one was intubated and died at 2 months, the other had a dilatation and is alive with disease at 10 months. In the eight patients who had a resection there was one post-operative death. Two patients died of metastatic disease at 6 and 8 months and one collapsed at home and died at 4 months. The remaining four are alive and disease free at 14, 10, 10 and 6 months.

Two patients had liver deposits at entry. Both these died at 3 months. One patient had lung metastases and died at 13 months. One patient with severe cardiopulmonary disease who was not fit for surgery remains alive with disease at 6 months.

Correspondence: H.R. Matthews, Regional Department of Thoracic Surgery, East Birmingham Hospital, Bordesley Green East, Birmingham B9 5ST, UK.

Received 22 May 1988.

**Table I** Dose modification in relation to renal impairment.

| *Creatinine clearance* ($ml\,min^{-1}$) | *Percentage of dose* |
|---|---|
| >60 | 100% |
| 40–59 | 75% |
| 30–39 | 50% |
| <30 | 0% |

**Table II** Dose modification in relation to platelet and white cell counts.

| *Platelets (nadir)* | *White cells (nadir)* | *Percentage of dose* |
|---|---|---|
| >150 | >4 | 120% |
| 100–149 | >4 | 110% |
| 75–99 | 3–3.9 | 100% |
| 50–74 | 2–2.9 | 90% |
| 25–49 | 1–1.9 | 75% |
| <25 | <1 | 50% |

**Table III** Characteristics of the carboplatin-treated population.

| *Characteristic* | | *No. of patients* |
|---|---|---|
| Entered | | 15 |
| Evaluable | | 14 |
| Median age 62 (range 49–72) | | |
| Male | | 15 |
| Performance scale at entry | WHO grade 0 | 13 |
| | WHO grade 1 | 2 |
| Site of tumour | lower oesophagus | 14 |
| | middle oesophagus | 1 |

Toxicity was mild and manageable. Nausea and vomiting WHO grade 1 or 2 was seen after 20 of the 29 courses assessed. Severe haematological toxicity WHO grade 3 was only seen in two patients. Nephrotoxicity was mild with only one patient having a WHO grade 1 elevation of his serum creatinine.

Adenocarcinoma of the oesophagus is increasing in incidence in both sexes but especially in males (Matthews *et al.*, 1987). Carboplatin appears to be ineffective as a single agent at a dose of $400\,mg\,m^{-2}$ in this disease. The drug is suitable for day-case administration and the toxicity is mild and manageable. Further studies of chemotherapy in this disease are required to prolong the survival of patients with inoperable disease and in those where resection is possible.

This study was funded by the Oesophageal Cancer Research Appeal (OCRA), Birmingham.

### References

GEHAN, E.A. (1961). The determination of the number of patients required in a preliminary and a follow up trial of a new chemotherapeutic agent. *J. Chron. Dis.*, **13**, 346.

KELSEN, D. (1984*a*). Chemotherapy of esophageal cancer, *Semin. Oncology*, **11**, 159.

KELSEN, D., STERNBERG, C., EINZIG, A. & 5 others (1984*b*). Phase II study of carboplatin (CBDCA) in advanced upper gastrointestinal tract (UGIT) malignancy. *Proc. Am. Soc. Clin. Oncol.*, **3**, 552 (*abstract*).

MATTHEWS, H.R., WATERHOUSE, J.A.H., POWELL, J., McCONKEY, C.C. & ROBERTSON, J. (eds). Cancer of the Oesophagus. *Clinical Cancer Monographs*, Vol. 1. The Macmillan Press 1987.

MILLER, A.B., HOOGSTRATEN, B., STAQUET, M. & WINKLER, A. (1981). *Reporting Results of Cancer Treatment. Cancer*, **47**, 207.

STERNBERG, C., KELSEN, D., DUKEMAN, M., LEICHMAN, L. & HEELAN, R. (1985). Carboplatin; a new platinum analog in the treatment of epidermoid carcinoma of the esophagus. *Cancer Treat. Rep.*, **69**, 1305.